# Functional Constipation and Dyssynergic Defecation in Children

**DOI:** 10.3389/fped.2022.832877

**Published:** 2022-02-16

**Authors:** Ilan J. N. Koppen, Marc A. Benninga

**Affiliations:** Department of Paediatric Gastroenterology and Nutrition, Emma Children's Hospital, Amsterdam UMC, University of Amsterdam, Amsterdam, Netherlands

**Keywords:** constipation, children, defecation, treatment, dyssynergia

## Abstract

Defecation is a complex physiological process, which relies on intricate mechanisms involving the autonomic and somatic nervous system, the pelvic floor muscles, and the anal sphincter complex. Anorectal dysfunction may result in constipation, a bothersome defecation disorder that can severely affect daily lives of children and their families. In this review, we focus on different mechanisms underlying anorectal dysfunction and specific treatment options aimed at improving defecation dynamics in children with functional constipation.

## Introduction

The act of defecation—the evacuation of feces—is a normal part of everyday life, occurring seemingly effortless from the moment we are born. However, the physiological processes that facilitate normal defecation are complex and rely on intricate mechanisms. These mechanisms relate to the autonomic and somatic nervous system, pelvic floor muscles, and the anal sphincter complex. Disturbances of these mechanisms may result in defecation disorders, which may affect both children and their parents. The most common defecation disorder in children is functional constipation (FC), occurring in ~10% of children worldwide (1). The term “functional” indicates that no organic cause can be identified in these children, this diagnosis is based on the Rome IV criteria (Table 1) (2, 3). In this review, we provide an overview of pathophysiological mechanisms underlying FC in children and specific treatment options aimed at improving defecation dynamics in children.

**Table 1:** Rome IV criteria for functional constipation for infants/toddlers and children/adolescents (2, 3).

**Infants/toddlers**
G7. Diagnostic criteria for functional constipation
Must include 1 month of at least 2 of the following in infants up to 4 years of age:
1.2 or fewer defecations per week
2. History of excessive stool retention
3. History of painful or hard bowel movements
4. History of large-diameter stools
5. Presence of a large fecal mass in the rectum
In toilet-trained children, the following additional criteria may be used:
6. At least 1 episode/week of incontinence after the acquisition of toileting skills
7. History of large-diameter stools that may obstruct the toilet
**Children/adolescents**
H3a. Diagnostic criteria for functional constipation
Must include 2 or more of the following occurring at least once per week for a minimum of 1 month with insufficient criteria for a diagnosis of irritable bowel syndrome:
1.2 or fewer defecations in the toilet per week in a child of a developmental age of at least 4 years
2. At least 1 episode of fecal incontinence per week
3. History of retentive posturing or excessive volitional stool retention
4. History of painful or hard bowel movements
5. Presence of a large fecal mass in the rectum
6. History of large diameter stools that can obstruct the toilet
After appropriate evaluation, the symptoms cannot be fully explained by another medical condition

## Physiology of Defecation

In this section we provide a brief summary of normal defecation dynamics, a thorough overview of these complex processes goes beyond the scope of this review but has recently been published by Heitmann et al. (4).

After passing the stomach, duodenum, jejunum and ileum, chyme is transported from the terminal ileum into the cecum. Here, it enters the colon. Subsequently, colonic contents are moved distally through the colon while gradual desiccation and mixing occurs and fluid is absorbed from the fecal mass. This results in more solid fecal material (5). The propulsion of feces is achieved by colonic motor patterns, which are able to move colonic contents in both antegrade and retrograde direction. Thus far, several different colonic motor patterns have been described, the most well-recognized patterns are high-amplitude propagating contractions (HAPCs) (6, 7). HAPCs are associated with antegrade mass movement of colonic contents and spontaneous defecation in healthy adults (8, 9). Forward propagation of feces through the colon leads to filling of the rectum, which induces involuntary relaxation of the internal anal sphincter via the recto-anal inhibitory reflex. This allows feces to travel further down the anal canal, where it comes in contact with the mucosa of the proximal part of the anal canal. This allows for “sampling” of the rectal contents in order to discriminate between solid, fluid and gas. These sensations in combination with sensory stimuli triggered by rectal distension result in an urge to defecate. At this point, voluntary contraction of the external anal sphincter and pelvic floor muscles can postpone defecation by moving the fecal load higher up in the rectum, until place and time are appropriate for defection. When defecation is initiated, voluntary relaxation of the external anal sphincter and the pelvic floor musculature (i.e., the puborectalis muscle and levator ani) facilitates the evacuation of stools via the anal canal. During defecation, a gentle increase in intra-abdominal pressure aids in expelling stools from the rectum.

Based on the physiological mechanisms described above, defecation can be divided into four phases (4). The basal phase is usually characterized by an empty rectum, partly due to retrograde motor patterns, a process known as the rectosigmoid brake. During the pre-expulsive phase, colonic contents are moved into the rectum, filling the rectum, activating the recto-anal inhibitory reflex and resulting in an urge to defecate. During the expulsive phase, expulsion of feces is facilitated by voluntary relaxation of the external anal sphincter and pelvic floor. Postural changes can help to reduce the anorectal angle, this can be achieved by taking a “squatting position” with the knees lifted above hip level and proper foot support. In the end phase, the anal canal pressure increases until it again exceeds the rectal pressure.

## Early Childhood Development and the Acquisition of Toileting Skills

The acquisition of toileting skills, defined as the process of establishing continence, is considered to be an important milestone in normal childhood development. Nowadays, toilet training is usually initiated between 21 and 36 months of age (10). By the age of 4 years, children have usually acquired bowel and bladder control (11). Interestingly, the age of onset of FC peaks around 1 year of age and decreases after the age of 4 years, with a median age of onset of 2.3 years (12). Thus, the most common defecation disorder in children has its peak incidence around the same age when toilet training occurs. When taking this into account, it is not surprising that behavioral factors are considered to play an important role in the development of childhood FC. One of the most important etiologic factors of childhood FC is stool withholding behavior. Withholding behavior often occurs after a negative experience such as a hard, painful or frightening bowel movement. It may also occur if children are preoccupied with other activities and therefore postpone defecation. Stool withholding behavior allows fecal matter to remain in the colon and rectum longer, where water is absorbed and the fecal mass becomes harder and more difficult to evacuate. This can eventually lead to the accumulation of a large fecal mass in the rectum, known as fecal impaction. If, after a period of withholding, a child needs to evacuate a hard, large diameter stool, this frequently results in painful defecation. This painful experience again prompts withholding behavior resulting in a vicious circle that can prove hard to break. In addition, fecal impaction may lead to overflow fecal incontinence which is the involuntary loss of soft stools that leak around the solid, obstructing, fecal mass. Fecal incontinence has been reported to be present in 75–90% of children presenting with FC and can have a detrimental effect on a child's and family's quality of life (13, 14).

## Withholding Behavior, Functional Outlet Obstruction or Dyssynergic Defecation: A Semantic Discussion?

Withholding behavior in children is common and therapeutic management is aimed at improving toileting behavior and facilitating frequent bowel movements. Laxatives are often prescribed to soften the stools and to prevent painful defecation. In the pediatric literature on constipation, a distinction is often made between slow transit constipation and functional outlet obstruction. Slow transit constipation indicates a slow transit of colonic contents throughout the entire colon (often defined as a colonic transit time >62 h). Functional outlet obstruction indicates that patients have a normal transit time through the colon but that feces accumulates in the rectum. A diagnosis of functional outlet obstruction suggests anorectal dysfunction as an underlying problem of constipation. In the adult literature, a number of terms have been used to describe constipation associated with anorectal dysfunction, these include the terms anismus, pelvic floor dyssynergia, obstructive defecation, paradoxical puborectalis contraction, pelvic outlet obstruction and spastic pelvic floor syndrome (15). Although the term “pelvic floor dyssynergia” is commonly used in the literature, it has been suggested that the term “dyssynergic defecation” most aptly describes this form of constipation. This is more specific and refers to the particular affected pelvic floor function (i.e., defecation), since urinary function and sexual function are often not impaired in these patients (15). The term dyssynergic defecation is used to describe the inability to coordinate the abdominal and pelvic floor muscles to evacuate stools due to paradoxical anal contraction or inadequate anal relaxation. Dyssynergic defecation is generally described after anorectal manometry or a balloon expulsion test. The balloon expulsion test is a reliable method to screen for pelvic floor dyssynergia in adults. This test is not commonly performed in young children (<5 years of age) because it requires a high level of patient cooperation (16, 17). The role of anorectal manometry in diagnosing dyssynergic defecation remains debatable. Several studies in adults have shown that the anal pressure may exceed the rectal pressure during simulated defecation in healthy volunteers undergoing anorectal manometry, which disputes that this manometric finding indicates pathophysiological defecation dynamics (4, 18).

An interesting difference between the pediatric literature and the adult literature, is that withholding behavior in children is often –at least in the beginning- considered a voluntary process based on a frightening or painful experience, while dyssynergic defecation in adolescents and adults is often considered a less voluntary and less conscious process.

A separate entity among anorectal dysfunctions in children is internal anal sphincter achalasia (IASA). Definitions of IASA vary in the literature and the entity is not well described (19). IASA is characterized by high anal pressures (hypertonic sphincter) in combination with the absence of a recto-anal inhibitory reflex on anorectal manometry but without evidence of aganglionosis on rectal biopsies. Another distinct group of children with constipation has high anal resting pressures while the recto-anal inhibitory reflex is present, some authors have suggested that this may be due to a reflexic overactivation of the external anal sphincter (20).

## Rectal Compliance and Hyposensitivity

Rectal compliance describes the relationship between rectal pressure and rectal volume, this reflects the ability of the rectum to act as a reservoir. To date, evidence on the role of rectal compliance in the pathophysiology of childhood FC is ambiguous. Although a higher rectal compliance has been described in FC patients compared to healthy volunteers, the relationship with clinical outcomes is unclear. For example, patients with FC in clinical remission have been reported to still have an increased rectal compliance and an increased rectal compliance is not associated with treatment failure (21–23).

Rectal compliance has also been suggested to potentially play a role in impaired rectal sensation (24). The hypothesis supporting this theory is that prolonged fecal stasis in the rectum could result in rectal dilation, altered distensibility of the rectal wall and an increased rectal compliance. These factors could result in visceral hyposensitivity (25). However, this theory has also been disputed in a study where no statistically significant differences in rectal sensitivity thresholds were identified between children with FC and healthy controls despite differences in rectal compliance (22).

## Conventional Treatment

Conventional medical management of childhood FC consists of non-pharmacological and pharmacological interventions (26).

### Non-pharmacological Management

Education is the first step in the non-pharmacological treatment of FC (27). This should include an explanation of physiological defecation dynamics, tailored to the developmental age of the child. There should be attention for a correct posture during toilet sits. A relaxed posture, with proper foot support and the knees lifted at or above hip level, helps to reduce the anorectal angle. The negative chain of events that may have been prompted by a painful defecation experience should be explained to parents and, if possible, children. Moreover, the concept of overflow incontinence and the pivotal role that withholding behavior plays in the pathophysiology need to be explained.

In order to prevent the occurrence of fecal impaction, it is important to evacuate stools regularly. In children with a developmental age of ≥4 years, this can be established by introducing a toilet training program, with scheduled toilet sit moments lasting 5 mins throughout the day. The toilet sit moments are scheduled after a meal to benefit from the colonic meal response which increases colonic peristalsis and tone in response to a meal. To motivate children to maintain this toilet training program, a reward system can be introduced. By rewarding the child for completing toilet sit moments, the child is positively reinforced to comply with therapy.

### Pharmacological Management

The pharmacological treatment of FC consists of treatment with laxatives and involves three steps; disimpaction, maintenance treatment and weaning. Prior to initiation of maintenance treatment, any potential fecal impaction needs to be evacuated in order to increase treatment success (13). Disimpaction can be achieved with high-dose oral polyethylene glycol (PEG) during 3–6 days or with enemas (28–30). PEG is poorly absorbed by the intestinal wall, causing osmotic water retention in the intestinal lumen, thereby softening the stools and increasing peristalsis. After successful disimpaction, maintenance therapy with PEG should be initiated to prevent the re-accumulation of feces (13). Maintenance treatment should be gradually weaned because abrupt cessation may induce a relapse (31). Weaning can be considered when symptoms are stable for at least one month under maintenance treatment, which means that children have a defecation frequency of ≥3 times per week and do not fulfill any other Rome criteria.

## Additional Non-Pharmacological Treatment Options

Despite pharmacological interventions ~40% of children with FC referred to a pediatric gastroenterologist still has symptoms after 5 years and even after 10 years 20% of children experience FC symptoms (26). In order to improve outcomes in this patient population, several pharmacological and non-pharmacological interventions have been investigated over the past years. In this section we will discuss non-pharmacological interventions aimed at improving defecation dynamics.

### Behavioral Therapy

It is assumed that behavioral play therapy can reduce phobic reactions related to defecation and that this may help to establish adequate toileting behavior (32). The learning process for children and their parents consists of five sequential steps: know, dare, can, will, and do (33). Children learn to recognize their urge to defecate and consequently it becomes a habit to use the toilet to evacuate sufficient amounts of feces instead of withholding it. One study, including 134 children, evaluated the effect of behavioral therapy by a psychologist as an add-on to laxative treatment and toilet training. In this study, treatment success rates and defecation frequency were not significantly different between groups. Therefore, there is currently no evidence for the routine addition of behavioral therapy in the treatment of childhood FC (32, 34). On the other hand, FC has been reported to be more common in children with specific behavioral disorders such as autism and attention deficit disorders (35, 36). These behavioral disorders are likely to influence withholding behavior. In children with behavioral disorders, behavioral therapy should therefore always be considered (26, 32).

### Biofeedback

A total of four studies, including 320 children, evaluated the effect of biofeedback in children with FC. Three studies reported on the effect of biofeedback as an add-on to laxative treatment compared with conventional treatment with laxatives (37–39). One study compared biofeedback at home and in the clinical setting—both as add-on treatment to laxative treatment (40). A recent meta-analysis, including the three studies comparing add-on treatment with biofeedback to conventional treatment, detected considerable levels of heterogeneity and found no evidence for benefit of the addition of biofeedback in children with FC (34). Interestingly, there is evidence that biofeedback training is beneficial in adult patients with FC and dyssynergic defecation (41, 42). Moreover, in the elderly population, sensory training in patients with abnormalities of rectal sensation is beneficial and can help to facilitate defecation. The differences in outcomes between pediatric and adult studies may rely on differences in the pathophysiology underlying pediatric and adult FC (43).

### Pelvic Physiotherapy

Several studies have evaluated the effect of pelvic muscle exercises as part of a combined treatment intervention, for example in addition to interferential electrical stimulation (44–46). Two studies have evaluated the effect of pelvic muscle exercises in addition to laxative treatment, compared with conventional treatment alone. One study performed in a clinical setting included 53 children and found that the treatment success rate was significantly higher in the group receiving additional pelvic physiotherapy (47). However, a large study in a primary care setting, including 134 children, did not find additional benefit of pelvic physiotherapy (48).

### Transanal Irrigation

Transanal irrigation is a treatment modality that can be used in children with FC who are unresponsive to pharmacological treatment. During transanal irrigation, a catheter or cone is inserted into the rectum to infuse water, thereby cleaning out the rectum and colon in a retrograde manner. The use of transanal rectal irrigation has been well established in patients with neurogenic bowel disorders and anorectal malformations (49, 50). Data on the efficacy of transanal irrigation in children with FC is limited but small pediatric cohort studies have suggested that transanal irrigation can be effective in the treatment of fecal incontinence and constipation symptoms and high parental satisfaction rates have been reported (51–55).

## Surgery

### Botox

If anorectal dysfunction or functional outlet obstruction is suspected, botulinum toxin A (botox) injections into the anal sphincter can be considered. Botox induces a temporary chemical paralysis of smooth and striated muscle fibers by blocking the release of acetylcholine from neurons. Several studies have shown that injection of botulinum toxin into the internal anal sphincter can be an effective treatment for children with FC (56–62). Interestingly, one study found that children with normal and abnormal sphincter dynamics based on anorectal manometry studies had similar responses to botox injections into the internal anal sphincter (61). Although less commonly reported in the literature, there have been reports of successful outcomes after injection of botulinum toxin into the external anal sphincter (63, 64).

### Rectal Prolapse Surgery

Rectal prolapse may develop as a consequence of prolonged straining in children with constipation. While in the majority of children, the rectal prolapse resolves spontaneously or as a consequence of medical management, a small but significant subset of patients may require surgery if the prolapse persists (65).

### Antegrade Continence Enemas

In addition to transanal irrigation, there are also surgical treatment options to achieve antegrade colonic irrigation, this is called antegrade continence enemas (ACE). ACE enables flushing fluid through the entire colon through an external opening into the colonic lumen, usually located at the cecum. The most well established procedures to facilitate ACE are the percutaneous cecostomy and the Malone appendicocecostomy. ACE surgery is considered minimally invasive and good clinical outcomes have been reported in children (60).

### Surgical Resections and Ostomies

In patients with severe symptoms, colonic resection or an ileostomy or colostomy can be considered. There are no clear guidelines on this type of surgical management for children with FC (60, 66). Due to the complex nature and the risk of complications, these interventions are usually performed in specialized centers in a multidisciplinary team.

## Discussion

Although withholding behavior is an important etiologic factor in the pathogenesis of FC, the terminology surrounding anorectal dysfunction can be confusing. The relevance of differentiating between patients with or without dyssynergic defecation remains unclear in the pediatric population. The accuracy of anorectal manometry as a diagnostic testing modality to make such a differentiation remains debatable and in children the balloon expulsion test is highly dependent on the level of cooperation. More importantly, unlike in the adult population, there is currently insufficient evidence to suggest that children with dyssynergic defecation would benefit from specific treatments such as biofeedback.

Still, there is evidence to support the important role of anorectal dysfunction in children with FC. For instance, studies on botox injections in the anal sphincter show that overcoming a functional outlet obstruction can result in favorable outcomes in children. Also, applying retrograde or antegrade colonic irrigation, with fluid volumes which are difficult to retain, has resulted in satisfactory outcomes.

The importance of withholding behavior in children has also been suggested as a potential explanation for the disappointing results of pediatric trials investigating novel pharmacological agents which have proven to be effective in adults, such as prucalopride and lubiprostone (67–69). Inclusion of children with severe withholding behavior may negatively affect study outcomes investigating pharmacological agents, if these agents do not target anorectal dysfunction. However, because withholding behavior is so common in children with FC, excluding such patients is also not desirable. Moreover, withholding behavior is included in the Rome IV criteria and excluding children with withholding behavior would therefore not provide a study sample representative of pediatric patients with FC. However, in future trials it could be worthwhile to account for potential differences in response rates between children with and without anorectal dysfunction.

In conclusion, withholding behavior plays an important role in the pathophysiology of FC in children. Unlike in the adult population, the clinical relevance of a diagnosis of dyssynergic defecation based on anorectal manometry or a balloon expulsion test remains debatable in the pediatric population. At the same time, anal botox injections aimed at overcoming a potential outlet obstruction seem promising, regardless of manometric findings prior to the intervention.

## Author Contributions

All authors listed have made a substantial, direct, and intellectual contribution to the work and approved it for publication.

## Conflict of Interest

The authors declare that the research was conducted in the absence of any commercial or financial relationships that could be construed as a potential conflict of interest.

## Publisher's Note

All claims expressed in this article are solely those of the authors and do not necessarily represent those of their affiliated organizations, or those of the publisher, the editors and the reviewers. Any product that may be evaluated in this article, or claim that may be made by its manufacturer, is not guaranteed or endorsed by the publisher.
